# Associations among quality of life, activities, and participation in elderly residents with joint contractures in long-term care facilities: a cross-sectional study

**DOI:** 10.1186/s12877-022-02870-6

**Published:** 2022-03-12

**Authors:** Yi-chang Chen, Keh-chung Lin, Shu-Hui Yeh, Chih-Hung Wang, Ay-Woan Pan, Hao-Ling Chen, Chen-Jung Chen

**Affiliations:** 1 Department of Rehabilitation, Jenteh Junior College of Medicine, Nursing and Management, 79-9, Sha-Luen Hu Xi-Zhou Li Hou-Loung Town, Miaoli County, Taiwan; 2 School of Occupational Therapy, College of Medicine, National Taiwan University, 17, F4, Xu-Zhou Road, Taipei, Taiwan; 3 Division of Occupational Therapy, Department of Physical Medicine and Rehabilitation, National Taiwan University Hospital, 7 Chung-shan South Road, Taipei, Taiwan; 4 Institute of Long-term Care, Mackay Medical College, 46, Sec. 3, Zhongzheng Rd., Sanzhi Dist, New Taipei City, Taiwan; 5 Graduate Institute of Education, National Changhua University of Education, 1 Jin-De Road, Changhua City, Taiwan; 6 Department of Nursing, Mackay Medical College, 46, Sec. 3, Zhongzheng Rd., Sanzhi Dist, New Taipei City, Taiwan

**Keywords:** Activity limitations, Elderly residents, Joint contractures, Osteoarthritis, Participation restrictions, Quality of life

## Abstract

**Background:**

Joint contractures and degenerative osteoarthritis are the most common joint diseases in the elderly population, can lead to limited mobility in elderly individuals, can exacerbate symptoms such as pain, stiffness, and disability, and can interfere with social participation and quality of life, thus affecting mental health. However, relevant studies on this topic are very limited. This study describes the associations of joint contracture categories and sites in elderly residents in long-term care facilities with their quality of life, activities, and participation.

**Methods:**

Elderly individuals with joint contractures who were residents in long-term care facilities were recruited. The World Health Organization (WHO) Quality of Life and the WHO Disability Assessment Schedule 2.0 were used to survey the participants. Correlations, multiple linear regressions, and multiple analyses of variance, with joint contractures as the response variable, were used in the statistical analysis.

**Results:**

The final statistical analysis included 232 participants. The explanatory power of contracture sites on activities and participation had a moderate strength of association (η^2^ = .113). Compared with elderly residents with joint contractures and osteoarthritis isolated to the upper limbs, those with joint contractures and osteoarthritis in both the upper and lower limbs had significantly worse activity and participation limitations. No significant differences in activity and participation were found between elderly residents with joint contractures affecting only the upper limbs and those with joint contractures affecting only the lower limbs (*F*_1,226_ = 2.604 and *F*_1,226_ = 0.674, nonsignificant). Osteoarthritis had the greatest impact on activity limitations and participation restrictions among elderly residents with joint contractures affecting both the upper and lower limbs (*F*_1,226_ = 6.251, *p* = .014).

**Conclusions:**

Elderly residents in long-term care facilities belonging to minority groups, with a history of stroke, and with osteoarthritis are at a high risk of developing activity limitations and participation restrictions. Moreover, compared with other contraction sites, regardless of osteoarthritis, joint contractures affecting both the upper and lower limbs were associated with the greatest activity limitations and participation restrictions.

**Trial registration:**

This study has been registered in the Chinese Clinical Trial Registry, registration number and date:ChiCTR2000039889 (13/11/2020).

**Supplementary Information:**

The online version contains supplementary material available at 10.1186/s12877-022-02870-6.

## Background

The rapid increase in global life expectancy indicates that joint contractures and osteoarthritis (OA), the most common joint diseases in the elderly population [1, 2], are becoming major global public health issues [3]. Joint contractures are present in more than 20% of elderly residents in long-term care facilities (LTCFs), and more than 10% develop symptoms of OA [1]. OA ranks fifth among all forms of disability worldwide [4]. Claims for data-based estimates consistently exceeded US $10,000 for individuals in the United States [5]. OA is the most common joint disease in the elderly population; it is a dynamic, pathologic, and chronic degenerative joint disease with a multifactorial aetiology and usually involves progressive structural destruction of key joints, progressive loss of articular cartilage, subchondral bone sclerosis, cyst formation, the development of osteophytes, and concomitant local low-grade inflammation [6, 7]. Although the definition of joint contracture currently lacks consensus [8], joint contracture is generally defined as a decrease in joint range of motion (ROM) due to various reasons [9, 10]. Joint contracture is classified into two categories, myogenic contracture and arthrogenic contracture [11, 12], and is characterized by a restricted joint ROM [13, 14].

Joint ROM limitation is an important factor for the development of joint contractures in the affected joints of many patients with OA [15], and joint contractures have long been regarded as one of the features of OA [16, 17]. Some scholars have proposed that the occurrence of joint contractures precedes OA. After OA develops, secondary capsular contractures may subsequently develop as complications, suggesting that the two may not share a causal relationship [14, 17]. Because the pathologic features of the two conditions are independent of each other, the occurrence of one condition is unlikely to lead to the occurrence of the other condition (causal relationship). However, the two conditions may be due to the same causes (e.g., immobility), and the occurrence site (regional) and the prevalence of these conditions are strongly negatively correlated, suggesting that some indirect implications between the two conditions remain to be clarified [14]. This finding indicates that the effects of joint contractures or OA on activity and participation, which are considered the most important factors affecting the quality of life (QoL) of elderly residents in LTCFs, may vary; that is, the QoL, activity, and social participation of individuals with joints affected by joint contractures or OA joints may differ from those of individuals with other conditions [18, 19].

Joint contractures are present in many elderly residents in LTCFs [1]. Joint contractures result in limited joint ROM [1, 20], which exacerbates OA-related symptoms, such as immobility, pain, stiffness, and disability, and accelerates OA progression [19]. Elderly individuals may experience substantial constraints in mobility and thus may experience activity and participation restrictions. Upper limb contractures may hinder independent eating, whereas lower limb contractures may restrict ambulation [21–24]. These limitations to activity may adversely affect the QoL of elderly residents in LTCFs [7, 25, 26] and result in disability among elderly residents and an increase in social expenditure [27]. In particular, elderly residents with joint contractures and OA have a higher likelihood of developing the abovementioned conditions than elderly residents with OA only [19]. However, few studies have focused on the effects of joint contractures and OA on QoL, activities, and social participation among the elderly population.

**Fig. 1:**
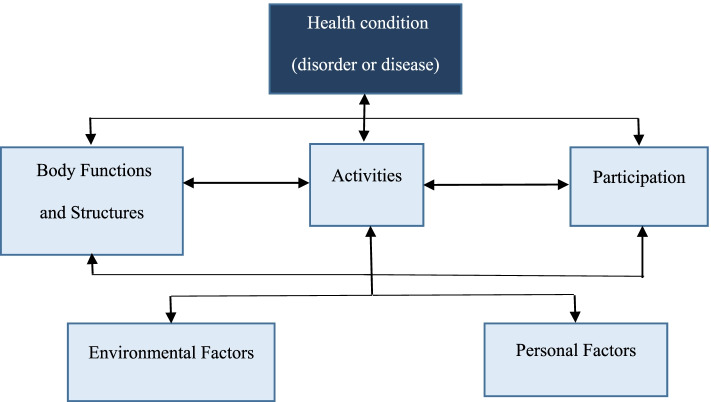
The ICF’s integrative model of functioning, disability and health

The International Classification of Functioning, Disability and Health (ICF) has become a global standard for the description and identification of disability [28]. The ICF has four components of health according to the WHO (2001). The first part, called Functioning and Disability includes the components “Body functions and structures” and “Activities and Participation”. For the second part, the Contextual Factors include the components “Environmental Factors” and “Personal Factors” that may affect the individual’s health status (Fig. 1) [29]. The biopsychosocial model of the ICF proposed by Engel [30] was adopted as the key theoretical conceptual model in the present study.

The basic assumption is that all diseases are the result of a complex interaction among biological, psychological, and social factors [30, 31], essentially treating the whole person as a biopsychosocial being [32]. This model provides a valuable overall reference framework for clinicians to consider the impact of psychological and social factors on an individual [33]. This model is currently becoming a dominant paradigm in psychiatry and psychosomatic medicine [34, 35] and has had an immense impact on medical education [33]. Engel argues that as a scientific framework, the biopsychosocial model can provide an adequate meta-framework to address the implicit philosophical framework challenges that medicine has been unable to address, allowing medicine to be positioned appropriately in empirical research programmes of different disciplines [33–35].

To prevent spread of the coronavirus disease 2019 (COVID-19), elderly residents with joint contractures in LTCFs must also adhere to the lockdown measures, and the limitations placed on travel may further reduce opportunities for activities and participation [36]. Based on clinical reasoning, we assumed that joint contractures and OA may negatively affect the ability of elderly individuals to protect themselves, such as wearing gloves and masks, cleaning surfaces, and washing hands, which may increase their risk of contracting and dying of COVID-19 [37]. Marks [7] predicted that the morbidity and mortality of elderly individuals with joint contractures or OA in LTCFs may increase. Therefore, these issues warrant attention because lockdowns and isolation can occur suddenly in any location during the COVID-19 pandemic. The purpose of the present study is to describe the associations of joint contracture categories (i.e., isolated contracture or contracture and OA) and contracture sites (i.e., isolated to the upper limbs, isolated to the lower limbs, or affecting both the upper and lower limbs) in elderly residents in LTCFs with their QoL, activities, and participation.

## Methods

### Design

This research was based on a cross-sectional study design.

### Setting and participants

Participants were recruited from May 2020 to July 2020 from registered LTCFs in Taiwan. A 3-stage stratified random sampling method was used to extract random samples with equal probability. The 3-stage sampling process was as follows:

1. Four LTCFs (nursing care facilities or elderly resident rehabilitation facilities) were selected from each of the northern, middle, and southern regions of Taiwan.
2. The person in charge of the LTCF that was selected for contact was interviewed by phone. After obtaining consent from the person in charge, the nursing chief in each facility screened for qualified residents. The investigators explained the research purpose to the eligible residents to obtain their informed consent.
3. A simple random sampling method was used to randomly select 25 residents from each list (the random numbers were generated by computers, and a total of 300 residents were selected from 12 LTCFs). Data were collected from August 2020 to October 2020, and the study investigators recruited the participants with the assistance of gatekeepers (e.g., nursing staff).

For sample size calculation, based on regression power analysis, the ratio between the explanatory variables and the number of samples should be 1:15–30. Because 11 explanatory variables were included in this study, 161 to 330 participants with joint contractures were needed to achieve a sufficient level of statistical power [38]. The study participants were residents of LTCFs, including public and private nursing homes, elder care and rehabilitation centres, retirement centres, and long-term care centres. The inclusion criteria were age ≥ 65 years residency in a long-term care facility for > 6 months, adequate language skills to complete or answer questionnaires, and at least one joint contracture site. Residents with cognitive dysfunction, major mental illnesses, and inflammatory arthropathies, such as seropositive arthropathies (rheumatoid arthritis or systemic lupus erythematosus), seronegative arthropathies (psoriatic arthritis or ankylosing spondylitis), crystal arthropathies (gout), and joint arthroplasty, were excluded.

### Measures

#### Disease-related and sociodemographic data

Based on the recommendation by interRAI, an international collaborative project, researchers used the Minimum Data Set (MDS) [39] tool to record sociodemographic data (e.g., sex and age). The criteria for determining cognitive status, joint contracture site, and OA were also defined in this study. The cutoff score for cognitive dysfunction was < 25 points on the Mini-Mental State Examination (MMSE). The MMSE results were obtained from the medical records of the participants. Participants without MMSE results completed the Chinese version of the Cognitive Abilities Screening Instrument (CASI C). The following outcomes were determined by the (CASI C): illiterate (years of education = 0, unable to read and write), < 50 points; some education (years of education = 1–5 years, some reading and writing skills), < 68 points; and educated (years of education = 6 years, able to read and write), < 80 points [40].

#### Measurement of joint contracture and osteoarthritis

The criterion for determining joint contracture categories (i.e., isolated contracture or contracture and OA) and contracture sites (i.e., isolated to the upper limbs, isolated to the lower limbs, or affecting both the upper and lower limbs) were confirmed by doctors, nurses, or therapists. The criterion for a contracture was a score of 3 on a 4-point scale (loss of > 2/3 of joint ROM) [41]. In a supine or lateral position, passive ROM of 12 main joints of both upper limbs (shoulders, elbows, and wrists) and both lower limbs (hips and knees) was measured using a goniometer and a 4-point scale [42]: 0 = normal (no loss of joint ROM); 1 = mild (loss of ≤1/3 of joint ROM); 2 = moderate (loss of ≤2/3 of joint ROM); and 3 = severe (loss of > 2/3 of joint ROM). The main advantage of the 4-point scale is that it can be used to measure ROM in a large number of joints within a limited time [41]. The interrater reliability of the 4-point scale was acceptable for the enrolled elderly residents with joint contractures in LTCFs, with a Kendall τ coefficient of 0.62 and bootstrapped 95% confidence interval (CI) of 0.49 to 0.74 [42].

The criterion for determining OA was radiographic evidence and involved physical examination by an experienced clinical orthopaedic specialist physician. Plain film radiographs are usually adequate for grading the joint space narrowing and size of osteophytes in accordance with the Radiological Indexes of the Osteoarthritis Research Society International (OARSI) atlas [43, 44]. Physical examination was used to assist in diagnosis in accordance with the International Classification of Diseases, Ninth Revision, Clinical Modification code: 715.xx [45].

#### The World Health Organization quality of life-BREF (WHOQOL-BREF)

The Chinese version of the WHOQOL-BREF, developed from the original version by the WHOQOL research headquarters, was used to evaluate the QoL of the participants [46, 47]. Twenty-six items are divided into 4 domains of physical health, psychological health, social relationships, and environment. Questionnaires with missing data exceeding 20% were discarded. Missing values were replaced by the average for the domain. If more than 2 values were in a domain, the domain score was not calculated (except for domain 3; the score was calculated if < 1 value was missing). The Cronbach α (internal consistency) for the entire questionnaire was .90, and the test-retest reliability for each domain was .75. The Pearson correlation coefficient between each item and the relevant domain ranged from .45 to .82 (*p* < .01), and the correlation among different domains ranged from .48 to .63 (*p* < .01). For the confirmatory factor analysis of construct validity, the structural equation model of the 4 domains echoed the designed potential structure of the questionnaire, and the comparative fitness index (CFI) of the 2 analyses was 0.886. These findings indicate that the Chinese version of the WHOQOL-BREF has good psychometric properties (reliability and validity) and is appropriate for use in Chinese-speaking people.

#### WHO Disability Assessment Scale 2.0 (WHODAS 2.0)

The 36 items of the World Health Organization (WHO) Disability Assessment Scale (WHODAS 2.0) were used to evaluate the activities and participation of the study participants, and the researchers used a 5-point Likert scale to determine the difficulties in the activities and participation. For scoring, the averages calculated according to the interpolation formula provided in the WHODAS 2.0 manual were used to replace missing data [48]. The score ranged from 0 (lowest difficulty) to 100 (maximum difficulty), and the score for each domain and the sum for the 6 domains were calculated. The higher the score was, the higher the degree of disability and the more severe the restriction. The severity of restrictions was determined based on the difficulty classification methods by the ICF and WHODAS 2.0. The classification of disability severity was as follows: below 4%, no disability; 5 to 24%, mild; 25 to 49%, moderate; 50 to 95%, severe; and greater than 96%, extremely severe [49]. The 4 items related to job ability were removed because all of the participants were retired or unemployed, and the remaining 32 items were used. For the Chinese version of WHODAS 2.0, the Cronbach α (internal consistency), a reliability index, was .73 to .99, and the intraclass correlation coefficient was .8 to .89 [50–52]. The Chinese version thus has excellent reliability and validity and is consistent with item response theory.

### Data collection procedures

A pre-test of the questionnaire was conducted with six residents from a nursing home in Taiwan. To explore the feasibility and acceptability of the questionnaire, a blank space was left below each item to allow those participating in the pre-test to provide questions and suggestions when completing the questionnaire. Health professionals who were not members of the research team underwent questionnaire and interview training and collected data using structured face-to-face interviews. Before each interview, the medical records of the participant were first examined to extract relevant sociodemographic data and diagnoses.

Each participant completed the questionnaire independently. If a participant was unable to complete the questionnaire independently due to vision, hearing, or reading or writing problems, the researchers provided assistance, for example, explaining certain sentences to ensure that the meaning was clear. The assistance provided by the researchers was consistent across participants; for instance, the examples provided were the same. If a participant was unable to answer a specific item, then the participant was allowed to ask the best-known person on site (relatives or nursing staff). Questionnaires with 50% or more information provided by relatives or nursing staff were marked, and these questionnaires were not used in the data analysis.

### Validity assurance and data analysis

When statistical surveys are used, missing items, rejection by respondents, researcher negligence, and issues related to the questionnaire itself can result in outliers and missing data; therefore, multiple imputation methods are used after a comparison with the original data [53]. Outliers were identified based on the Mahalanobis distance [54], and if 10% of the questionnaire comprised outliers and missing data, the questionnaire was excluded. Before the formal analysis, frequency analysis was performed on each variable to minimize operational errors during data input. Descriptive statistics were used to characterize the study participants.

All participants were stratified according to contracture category and contracture site. The mean, standard deviation (SD), and eta (η) correlation ratio were used for continuous variables (e.g., activities and participation and QoL). Absolute and relative frequencies and the Spearman rank order correlation coefficient (*r*_s_) were used for discrete variables, including ordinal variables such as education and number of visits for relatives and friends. The Pearson chi-squared (χ^2^) test was used for categorical variables (e.g., sex and marital status).

The Durbin-Watson statistical test was conducted to determine whether an autocorrelation existed between residuals. By using the ‘enter’ method to conduct multiple linear regression analyses after dummy coding categorical variables, the possible associations between the response variables (contractures affecting participants in different groups) and explanatory variables (demographic data, QoL, and activities and participation) were further investigated. Only the significant variables in the regression analysis were included in the bivariate analysis [51]. The 95% CI was used, and the significance level of each statistical test was set as *p* < .050.

Multiple analysis of variance (MANOVA) was used to detect differences in QoL and activities and participation among subjects in different groups. Finally, the interaction effect between variables was tested. The explanation of a main effect of a significant variable must consider the interaction of 2 independent variables; therefore, the main effect was not analysed, but the simple main effect test was conducted to compare the difference in activities and participation between category and sites. SPSS 20.0 software (IBM Corp, Armonk, NY, USA) was used for data processing and analyses.

### Ethics approval and consent to participate

All methods were carried out in accordance with the Declaration of Helsinki. The study was approved by the Institutional Review Boards of MacKay Memorial Hospital (IRB number: 14MMHIS177) and National Taiwan University (IRB number: 201905Hm137). Before the study, the study process was explained in detail to the participants, and the study was performed only after written informed consent was obtained from each participant. The data collected from the participants that may be queried, such as name and lifestyle, were deidentified and kept confidential. The data were used only for purposes of the study. Study participants were assured that they could withdraw at any time from the study without any consequences.

## Results

### Recruitment and participant characteristics

**Fig. 2:**
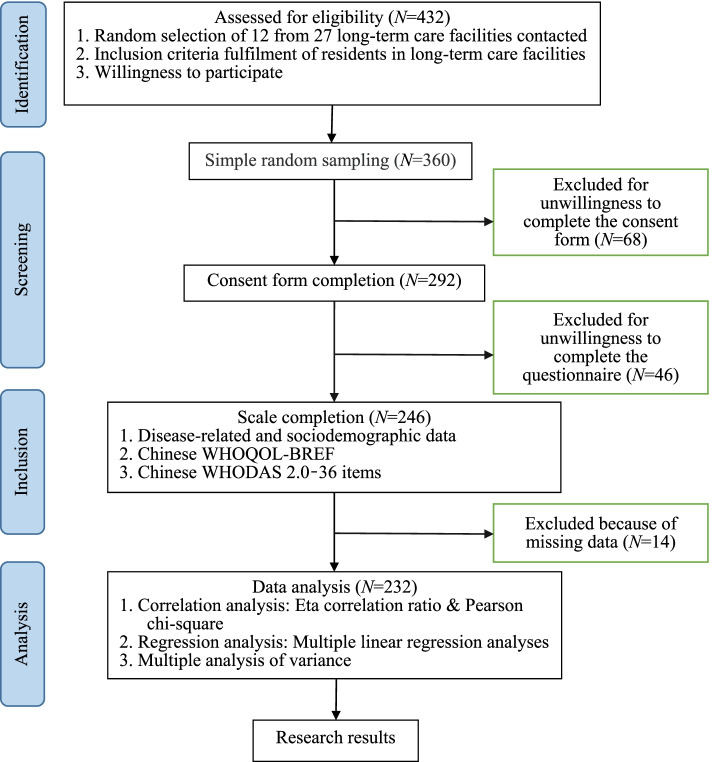
Flow diagram of participant selection for the study. LTC: long-term care; WHOQOL-BREF: The World Health Organization Quality of Life-BREF; WHODAS 2.0: The World Health Organization Disability Assessment Schedule 2.0

Of the 27 LTCFs contacted, 12 agreed to participate in this study. The other 15 care facilities refused to participate for various reasons, including staff shortages, lack of interest, and difficulties regarding coordinating research projects. From these 12 care facilities, 432 elderly residents met the inclusion criteria, 298 were selected by simple random sampling, and 246 completed the questionnaire, for a response rate of 82.55%. After 14 questionnaires with more than 10% missing data were excluded, 232 participants were included in the final statistical analysis. Figure 2 shows the detailed flow diagram of participant selection for the study based on the Strengthening the Reporting of Observational Studies in Epidemiology (STROBE) guidelines. The average age of the participants was 75.70 ± 9.93 years, and 56.5% were men. Among the participants, 176 (75.9%) had at least 1 isolated contracture, and 56 (24.1%) had at least 1 site with both a contracture and OA. Joint contractures in 105 participants (45.3%) were isolated and affected at least 1 lower limb, 82 (35.3%) had joint contractures affecting both the upper and lower limbs, and 45 (19.4%) had isolated joint contractures affecting at least 1 upper limb. The 4 most common chronic diseases were hypertension (29.7%), stroke (24.1%), cardiovascular diseases (23.3%), and diabetes (21.1%) (Table 1).

**Table 1:** Demographic and condition-specific characteristics of the participants (*N* = 232)

Characteristics	Mean ± SD (min–max)
Age (years)	75.703 ± 9.933 (65–98)
Length of residency (month)	37.517 ± 43.479 (6–240)
Body mass index (kg/m^2^)	22.320 ± 3.479 (12.11–34.67)
Characteristics	No. (%)
Contracture category	
Isolated contracture	176 (75.9)
Both contracture and OA	56 (24.1)
Contracture sites	
Isolated to the upper limbs	45 (19.4)
Isolated to the lower limbs	105 (45.3)
Both the upper and lower limbs	82 (35.3)
Sex	
Female	101 (43.5)
Male	131 (56.5)
Ancestry/ethnicity	
Min Nan	201 (86.6)
Hakka	8 (3.4)
Aborigines	3 (1.3)
Mainland Chinese	9 (3.9)
Others	11 (4.7)
Education	
Primary and below	122 (52.6)
Junior high	49 (21.1)
Senior secondary	30 (12.9)
Higher	12 (5.2)
College/university and above	19 (8.2)
Marital status	
Married	63 (27.2)
Widowed	82 (35.3)
Divorced	23 (9.9)
Single	64 (27.6)
Number of visits for relatives and friends (weeks)	
0–1	163 (70.2)
2–3	52 (22.4)
4–5	6 (2.6)
6–7	11 (4.7)
Chronic diseases	
Hypertension	69 (29.7)
Diabetes	49 (21.1)
Stroke	56 (24.1)
Cardiovascular disease	54 (23.3)
Cataract	4 (1.7)
Glaucoma	1 (.4)

*SD* standard deviation, *OA* osteoarthritis. Values are the mean and SD or percentages for continuous variables; values are the numbers and relative frequencies (%) for categorical variables

### Correlations among demographic characteristics, QoL, and activities and participation

A significant correlation was found between contracture category and the following variables: age (η = .176, *p* = .007), length of residency (η = .123, *p* = .030), sex (χ^2^ = 5.56, *p* = .018), stroke (χ^2^ = 14.22, *p* < .001), and cataracts (χ^2^ = 12.79, *p* < .001). The contingency coefficients for sex, stroke, and cataracts were 0.153, 0.240, and 0.229, respectively, indicating that these 3 factors and the 2 contracture categories (isolated contracture and both contracture and OA) had a significantly weak strength of association. A significant correlation was found between contracture site and the following variables: ancestry/ethnicity (χ^2^ = 24.07, *p* = .002), diabetes (χ^2^ = 6.08, *p* = .048), stroke (χ^2^ = 14.02, *p* = .001), and activities and participation (η = .277, *p* < .001). The contingency coefficients for ancestry/ethnicity, diabetes, and stroke were 0.307, 0.160, and 0.239, respectively, indicating that the 3 factors and the 3 types of contracture sites (isolated to the upper limbs, isolated to the lower limbs, and affecting both upper and lower limbs) had a significantly weak strength of association (Table 2).

**Table 2:** Correlations among demographic characteristics, quality of life, and activities and participation (*N* = 232)

	Contracture category				Contracture sites				
Variables	η	*r*_s_	χ^2^	*p*	η	*r*_s_	χ^2^	*p*	
Age (years)	.176**			.007	.086			.381	
Length of residency (months)	.143*			.030	.065			.382	
Body mass index	.006			.924	.123			.326	
Sex			5.561*	.018			1.733	.420	
Ancestry/ethnicity			4.055	.399			24.066**	.002	
Education		.036		.581		−.010		.882	
Marital status			0.593	.898			9.180	.164	
Visiting frequency (weeks)		.043		.510			−0.083	.209	
Chronic diseases									
Hypertension			0.505	.220			0.418	.811	
Diabetes			1.130	.288			6.082*	.048	
Stroke			14.219***	<.001			14.020**	.001	
Cardiovascular disease			3.250	.071			2.095	.351	
Cataract			12.792***	<.001			3.424	.180	
Glaucoma			0.320	.572			4.174	.124	
Activities and participation	.035			.599	.277***			<.001	
Quality of life	.078			.235	.101			.134	

η = eta correlation ratio; *r*_s_ = Spearman’s rank order correlation coefficient; χ^2^ = Pearson chi-squared statistic. *p < .05; **p < .01; ***p < .001, two-tailed

### Factors associated with activities and participation

For the multiple linear regression analysis, only the significantly correlated variables (Table 2) were included. The Durbin-Watson statistic was 1.625, indicating no remaining autocorrelation in residuals. The set of 17 main effects was significant (*F*_17, 214_ = 6.916, *p* < .001), and for the proportion of the variance that can be explained by the activity and participation of elderly residents, the multiple determination coefficient *R*^2^ was 0.355 (model 1), which was higher than the *R*^2^ (> 0.208) for the logistic regression [55, 56]. The condition index (condition number) was 34.239, indicating that collinearity was reduced [38]. The following variables were significantly related to activities and participation: ancestry/ethnicity (other) (β = −.169, *t* = − 2.802, *p* = .006), stroke (yes) (β = .270, *t* = 4.456, *p* < .001), and contracture category (both contracture and OA) (β = .214, *t* = 3.451, *p* = .001), indicating that these 3 factors were the most critical factors affecting activities and participation and collectively explain 29.8% of activities and participation (model 2).

Individual independent variables were further tested, and regression coefficients indicated that stroke had the best explanatory power (i.e., stroke can cause activity limitations and participation restrictions). The tolerance and variance inflation factor (VIF) statistics decreased to 0.922 and 1.085, respectively, indicating no significant collinearity (Table 3).

**Table 3:** Multiple linear regression analysis of factors associated with activities and participation (*N* = 232)

Factors	SE (B)	Beta (β)	*t* value	*P*	95% CI	
					Lower	Upper
(Constant)	78.504		4.130***	<.001	41.035	115.973
Age (years)	0.032	.010	0.138	.890	− 0.575	0.362
Length of residency (months)	0.025	.032	0.549	.584	−0.064	0.113
Sex						
Male	1.004	.015	0.247	.805	−7.011	9.020
Ancestry/ethnicity						
Hakka	0.032	.000	0.003	.998	−21.863	21.927
Aborigines	−6.296	−.021	−.369	.712	−39.924	27.331
Mainland Chinese	−17.841	−.104	−1.665	.097	−38.965	3.283
Other	−26.389	−.169	−2.802**	.006	−44.956	−7.823
Diabetes						
No	−5.709	−.070	−1.208	.228	−15.027	3.608
Stroke						
Yes	20.988	.270	4.456***	<.001	11.705	30.272
Cataract						
Yes	−31.027	−.122	−1.901	.059	−63.200	1.145
Contracture category						
Both contracture and OA	16.593	.214	3.451**	.001	7.117	26.069
Contracture sites						
Isolated to the upper limbs	−9.920	−.118	−1.854	.065	−20.468	0.629
Both the upper and lower limbs	5.575	.080	1.238	.217	−3.303	14.453
Model 1 (17 total variables)	*R*^*2*^ *=* .355		*adj R*^*2*^ *=* .303		*F*_(17,214)_ = 6.916, *p* < .001	
Model 2 (5 significant variables)	*R*^*2*^ = .298		*adj R*^*2*^ = .282		*F*_(5,226)_ = 19.166, *p* < .001	

*Beta (β)* standardized regression coefficient, *SE* standard error, *CI* confidence interval. *p < .05; **p < .01; ***p < .001, two-tailed

### Comparison of the differences in QoL and activities and participation between categories and sites

MANOVA was conducted to compare the differences between categories and sites with respect to QoL and activities and participation. For QoL (*F* = 2.117, *p* = .064) and activities and participation (*F* = 1.420, *p* = .218), the results for Levene’s test of homogeneity of variance were not significant, indicating no significant difference in the dispersion of samples between these 2 classifications (i.e., category and site). By contrast, for the elderly residents, statistically significant differences (Wilks λ = .882, *F*_2,226_ = 14.444, *p* < .001) were observed for activities and participation among the 3 contracture sites (isolated to the upper limbs, isolated to the lower limbs, and both the upper and lower limbs), indicating that residents with different contracture sites had significant differences in activities and participation. The post hoc Tukey honestly significant difference test for the 3 contracture sites and activities and participation indicated significant differences with respect to contractures isolated to the upper limbs vs. affecting isolated to the lower limbs (95% CI, − 28.109 to − 1.511; *p* = .025) and isolated to the upper limbs vs. affecting both the upper and lower limbs (95% CI, − 39.674 to − 11.980; *p* < .001).

**Fig. 3:**
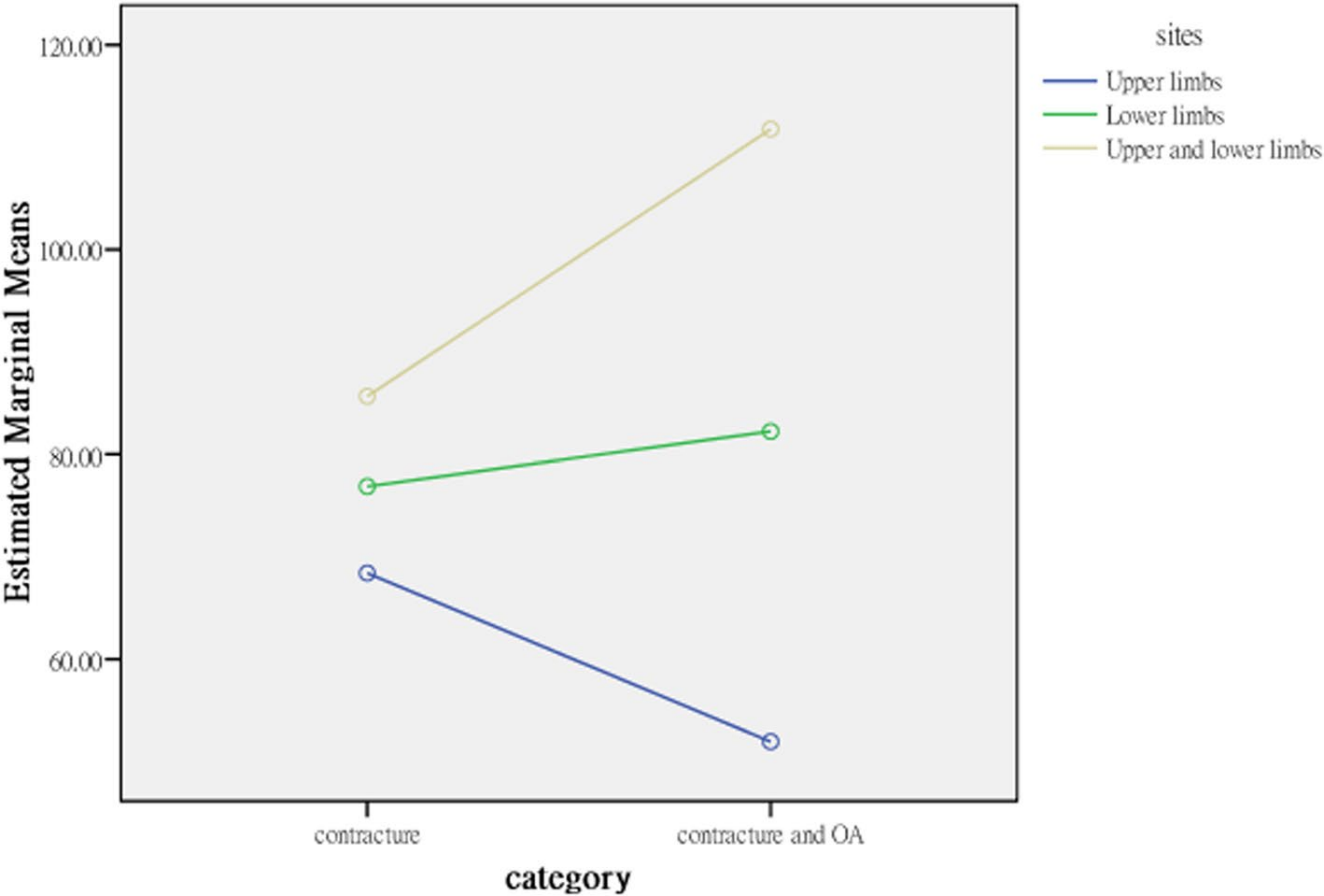
Interaction effect, activities and participation × contracture category

**Fig. 4:**
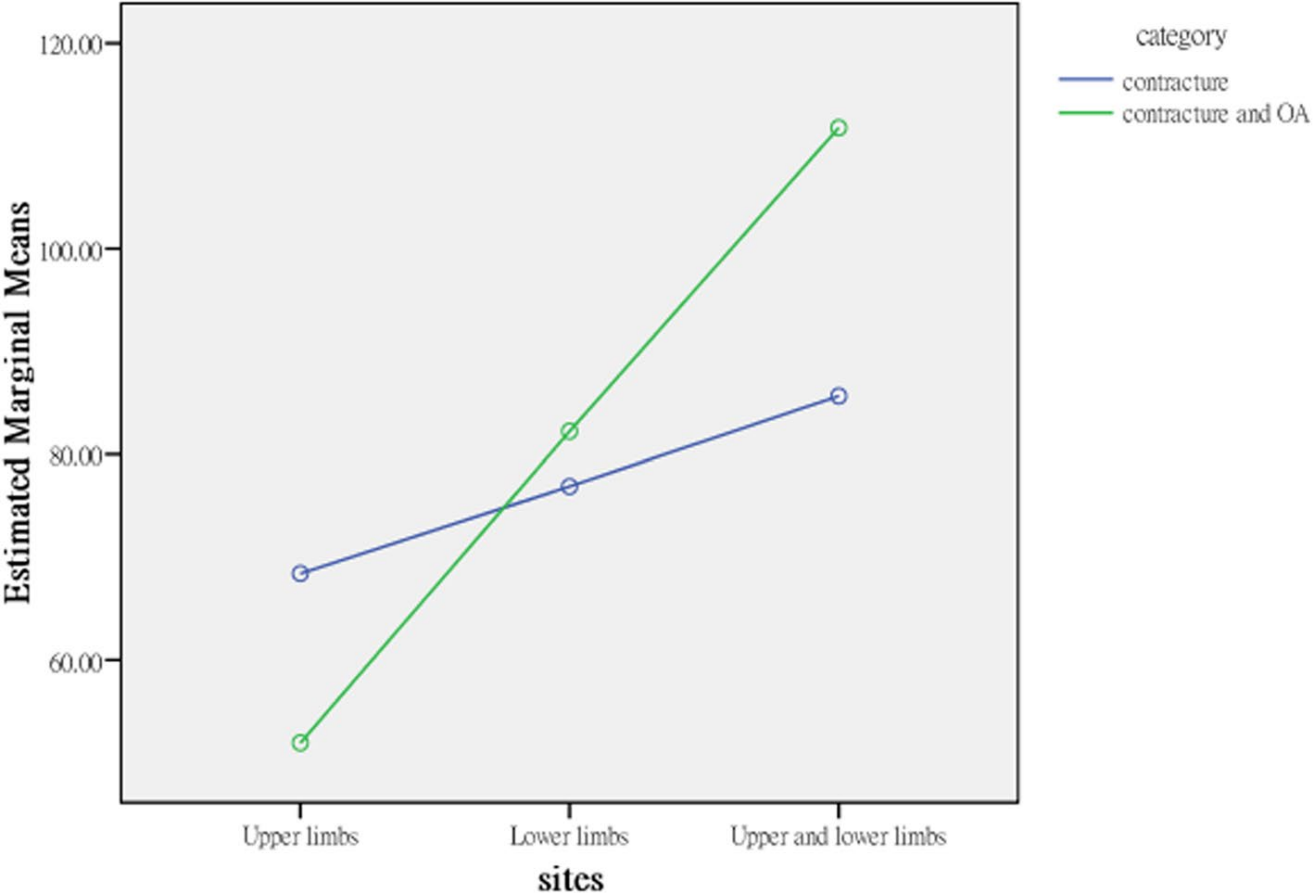
Interaction effect, activities and participation × contracture site

**Fig. 5:**
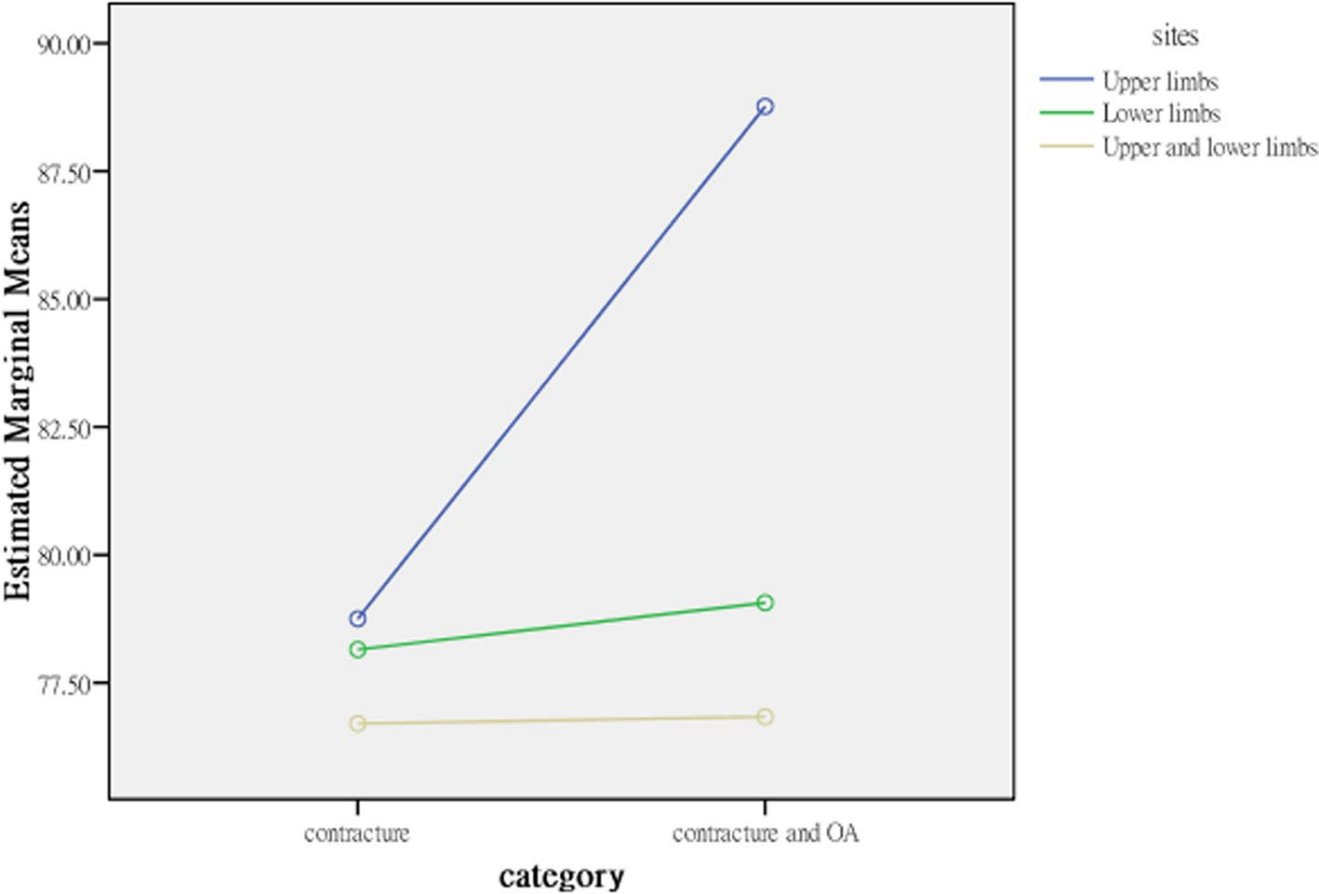
Interaction effect, quality of life × contracture category

**Fig. 6:**
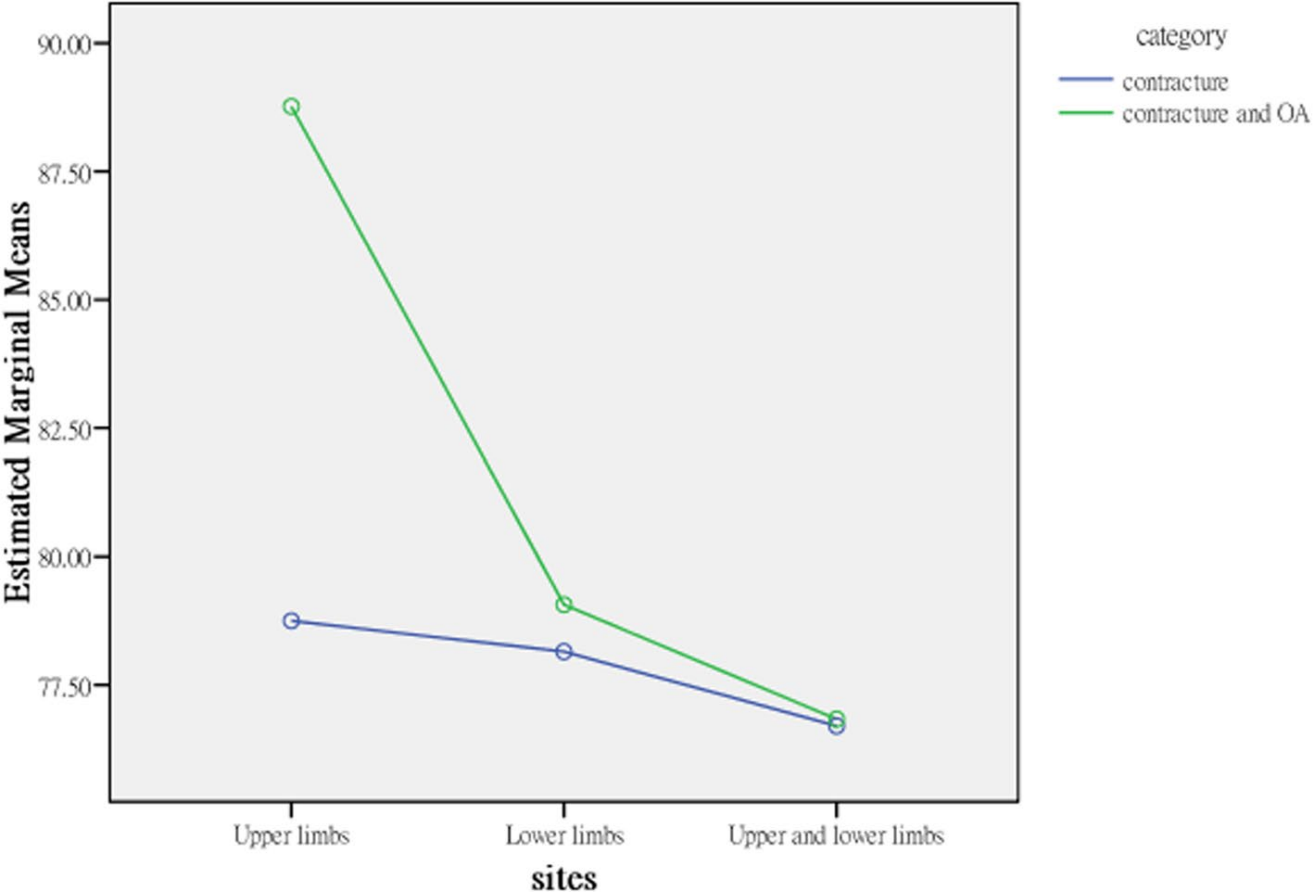
Interaction effect, quality of life × contracture site

The average activity and participation scores for patients with contractures isolated to the upper limbs (60.167) and isolated to the lower limbs (76.880) were significantly lower than that for both the upper and lower limbs (98.714), indicating that elderly residents with joint contractures affecting both the upper and lower limbs and contractures isolated to the lower limbs had more activity limitations and participation restrictions than those with joint contractures isolated to the upper limbs. The explanatory power of contracture site for activities and participation was 11.3% in terms of the η^2^ coefficient; therefore, the strength of the association was moderate (Table 4). Contracture category and site affect activities and participation interactively; the profile plots (Figs. 3, 4, 5, 6) clearly show an interaction effect and that the interaction effect was significant. Therefore, a test of the simple main effect was conducted to discuss under what circumstances activities and participation increase or decrease.

**Table 4:** MANOVA comparing differences between categories and sites (*N* = 232)

Sources of variation	Activities and participation								Quality of life			
	SS	*df*	MS	*F*	*p*	η^2^	SS	*df*	MS	*F*	*p*	η^2^
Contracture category (A)	891.879	1	891.879	.891	.346	.004	486.995	1	486.995	1.630	.203	.007
Contracture sites (B)	28,912.809	2	14,456.404	14.444***	<.001	.113	1040.411	2	520.205	1.741	.178	.015
A*B	8826.055	2	4413.028	4.409*	.013	.038	632.126	2	316.063	1.058	.349	.009
Error	226,198.890	226	1000.880				67,525.910	226	298.787			
Corrected Total	255,908.106	231					69,177.272	231				

*SS* Type III sum of squares, *df* degree of freedom, *F* F ratio, MS mean square, *η*^*2*^ partial eta squared. *p < .05; ***p < .001

### Simple main effect test to compare the difference in activities and participation between categories and sites

Before testing for the main effect based on analysis of variance (ANOVA), we used the family-wise error rate, α_FW_, to avoid expansion of type I errors. The α_FW_ of each test was set at α/5 = .05/5 = .01 to control the overall type I error at the .05 level. For elderly residents with joint contractures affecting both the upper and lower limbs but without OA (i.e., isolated contracture), the average for activity limitations and participation restrictions was 85.652, and for elderly residents with joint contractures and OA, the average for activity limitations and participation restrictions was 111.775, a result that was higher than that for residents with contractures isolated to the lower limbs. For those with contractures isolated to the upper limbs and with both joint contractures and OA, the average for activity limitation and participation restriction was 51.923, and for those with contractures isolated to the upper limbs and without OA (i.e., isolated contractures), the average for activity limitation and participation restriction was 68.410, but the increase was not significant.

The interaction effects of contracture category and contracture site on activities and participation were different in different groups. First, the effects of different contracture sites on the activities and participation of residents with joint contractures were analysed from the split file of 2 groups from the contracture category. In the isolated contractures group (i.e., without OA), the activities and participation of residents with joint contractures at different sites were significantly different (*F*_2,226_ = 3.311, *p* = .039). The post hoc test results found that activity and participation in residents with joint contractures affecting both the upper and lower limbs (*M* = 85.652) were significantly more restricted than those of residents with joint contractures isolated to the upper limbs (*M* = 68.410) but were not significantly more restricted than those of residents with joint contractures isolated to the lower limbs (*M* = 76.880). Pairwise comparisons showed that only the comparison between joint contractures isolated to the upper limbs and both the upper and lower limbs was significant. In addition, in the contracture and OA group, the effect of contracture site on activity limitations and participation restrictions was not only significant but also increased (*F*_2,226_ = 13.799, *p* < .001). The post hoc test results indicated that the activity limitations and participation restrictions of residents with joint contractures affecting both the upper and lower limbs (*M* = 111.775) was significantly higher than those of residents with joint contractures isolated to the lower limbs (*M* = 82.223) and residents with joint contractures isolated to the upper limbs (*M* = 51.923). The pairwise comparisons were all significant.

The effect of OA (i.e., contracture category) on the activities and participation of residents with joint contractures was analysed from the split file of 3 contracture site groups. In the participants with joint contracture isolated to the upper limbs and those isolated to the lower limbs, OA had no significant effect on the activities (*F*_1,226_ = 2.60) and participation (*F*_1,226_ = .674) of these residents (*F*_1,226_ = 2.604). However, in the participants with contractures in both the upper and lower limbs, OA had a significant effect on the activities and participation of these residents (*F*_1,226_ = 6.251, *p* = .014) (Table 5). Because the simple main effect of contracture category involves only 2 levels, a post hoc test was unnecessary, and the average scores of the 2 groups were compared.

**Table 5:** Simple main effects in the ANOVA comparing differences between categories and sites (*N* = 232)

Sources of variation	Activities and participation					
	SS	*df*	MS	*F*	*p*	Post hoc tests
Contracture sites						
Within isolated contracture	7013.515	2	3506.758	3.311*	.039	C > B, C > A, B > A
Within both the contracture and OA	22,387.676	2	1058.981	13.799***	<.001	C > B, C > A, B > A
Contracture category						
Within the upper limbs only (A)	2512.912	1	2512.912	2.604	.114	
Within the lower limbs only (B)	623.666	1	623.666	.674	.413	
Within both the upper and lower limbs (C)	6990.654	1	6990.654	6.251*	.014	
Error	226,198.890	226	1000.880			

*SS* Type III sum of squares, *df* degree of freedom, *F* F ratio, *MS* mean square. *p < .05; ***p < .001

## Discussion

Regardless of OA, joint contractures affecting both the upper and lower limbs were associated with the greatest activity limitations and participation restrictions compared with upper limb or lower limb contractures. However, there was no significant association between the type of joint contractures and QoL among the elderly residents.

For contracture category, differences in age, length of residency, sex, stroke, and cataracts were found between residents with contractures and OA and those with contractures without OA. This finding supports the notion by Campbell, Trudel, Wong, and Laneuville [57] that the gene signature is related to the sites of joint contracture combining OA rather than isolated OA without contractures. Two issues warrant consideration. First, because 5 variables were evaluated, some indirect implications may support the argument that no causal relationship exists between contractures and OA [14]; namely, contractures and OA (pathologic characteristics) may occur independently, and the occurrence of one condition is unlikely to lead to the occurrence of the other condition (causal relationship). However, even in the absence of a causal relationship, OA promotes the development of joint contractures due to its association with immobility (as an intermediate factor) [14, 58, 59]. Clinically, a period of immobility after trauma or decreased activity secondary to OA in predisposed individuals may show more collagen content in contracture capsules and lead to fibrotic changes in the posterior capsule of the knees, subsequent capsular shortening, and loss of full knee extension [57]. Overall, these results suggest a role of capsule fibrosis associated with OA and that some signature (e.g. genetic factors) is related to joint contracture and OA.

Second, the results of this study showed significant differences in activities and participation between the 2 groups of elderly residents with joint contractures (i.e., elderly residents with and without OA affecting both the upper and lower limbs), indicating that compared with elderly residents without OA affecting both the upper and lower limbs, elderly residents with OA affecting both the upper and lower limbs had significantly more activity limitations and participation restrictions, which is consistent with the findings of Steultjens et al. [19]; that is, for elderly residents in LTCFs, the effects of joint contractures and OA on QoL and activities and social participation may be different.

Third, after a detailed analysis, an interesting finding was no significant differences in the effect of contracture category (i.e., with and without OA) on QoL, activities, and social participation among residents; however, different contracture sites (i.e., isolated or both the upper and lower limbs) had significantly different effects on activities and social participation among residents. The underlying mechanisms can be discussed with the biopsychosocial model of the ICF: Some underlying diseases promote contracture development (i.e., health conditions). The severity of the body’s function and structure impairment is strongly related to the severity of activity limitations and participation restrictions regardless of the underlying disease (e.g., OA). Personal and environmental factors (e.g., assistive devices) can have a positive impact on the performance of activities and participation despite existing disabilities, which should also be discussed along with clarification of the underlying mechanisms in future studies.

Finally, this study found that time factors (i.e., age and length of residency) had different influences on the risk of OA in the 2 groups of elderly residents with joint contractures in LTCFs. In other words, older age and longer residency in a facility corresponded to a higher risk of OA, which indirectly corroborates results from previous studies [15, 60]. Therefore, measures to minimize the effects of time factors associated with joint contractures and OA, such as avoiding prolonged joint immobility, are particularly important.

For contracture site, the results showed differences in ancestry/ethnicity, diabetes, stroke, activities, and participation among the 3 groups of elderly residents. First, the results from this study not only support the previous finding that joint contractures affecting both the upper and lower limbs have a major impact on activities and participation [20] but also indicate significant differences in activity limitations and participation restrictions in elderly residents with joint contractures when comparing individuals with contractures isolated to the upper limbs vs. those isolated to the lower limbs and individuals with contractures isolated to the upper limbs vs. those affecting both the upper and lower limbs. However, no significant differences in the activities and participation of elderly residents with joint contractures were found when comparing residents with contractures isolated to the lower limbs vs. contractures affecting both the upper and lower limbs.

Comparing the results of this study to the findings of Bartoszek et al. [21] revealed no significant differences in activities and participation among patients with joint contractures when comparing those with contractures isolated to the upper limbs, isolated to the lower limbs, or affecting both upper and lower limbs, although consistency and differences were both observed. Regarding consistency, the same level of restriction on activities and participation was observed in the 2 groups of elderly residents with joint contractures isolated to the lower limbs vs. those affecting both the upper and lower limbs, while regarding differences, varying degrees of restrictions on activities and participation were noted in the groups of elderly residents with joint contractures. The reason for the differences may be that the previous study did not actively verify the joint contracture diagnosis; therefore, validity was not absolutely certain, and this limitation may be the main cause of the final conclusion indicating no significant differences.

Second, this study found that the most critical factors affecting the activities and participation of elderly residents in LTCFs were ancestry/ethnicity (other), stroke (yes), and contracture category (both contracture and OA), which can collectively explain nearly one-third (28.2%) of the variance in activities and participation among elderly residents with joint contractures in LTCFs. Therefore, minority status, stroke, and OA were the 3 key risk factors for joint contractures in elderly individuals residing in LTCFs. Minority status is a risk factor that warrants consideration. Residents who are of minority status in language and cultural identity may encounter barriers to interpersonal interaction and social exchanges. These barriers may impede their improvement and participation in activities and quality of life.

Finally, the results showed that the explanatory power of contracture site for activities and participation had a moderate strength of association (η^2^ = .113). Compared with other contraction sites, regardless of OA, joint contractures affecting both the upper and lower limbs were associated with the greatest activity limitations and participation restrictions.

The present study has 3 main findings. First, for the 3 groups of elderly residents with joint contractures and without OA (i.e., isolated to the upper limbs, isolated to the lower limbs, and affecting both the upper and lower limbs), the difference in activities and participation between the elderly individuals in only 2 groups (isolated to the upper limbs vs. affecting both upper and lower limbs) was significant. In other words, compared with residents with joint contractures isolated to the upper limbs but without OA, elderly patients without OA but with joint contractures affecting both the upper and lower limbs had significantly more activity limitations and participation restrictions.

Notably, it generally seems that impaired lower limb function has a greater impact on daily life. However, the results show that the difference in activities and participation between the elderly individuals with contractures isolated to the upper limbs and those with contractures isolated to lower limbs was not significant. A possible explanation is that the target population of the present study was residents of LTCFs rather than residents in the community. The main difference is that activities and participation of residents of LTCFs are commonly restricted to a sitting position for safety concerns. Residents with contractures isolated to the lower limbs may sit in a wheelchair to participate in upper limb activities. In contrast, residents with contractures isolated to the upper limbs are restricted in upper limb movements that are important for daily life activities. Residents with contractures isolated to the upper limbs and those with contractures isolated to the lower limbs encounter different barriers to their movement in daily life activities. However, both groups are restricted in functional movement in daily life activities. This may partly explain why no significant differences were found in activities and participation in these 2 groups of residents.

Second, pairwise comparisons of the 3 groups of elderly residents with joint contractures and OA (i.e., contractures isolated to the upper limbs, contractures isolated to the lower limbs, and contractures affecting both the upper and lower limbs) showed significant differences in activity performance and participation among the 3 groups. Compared with the residents with OA and joint contractures isolated to the upper limbs, elderly patients with OA and with joint contractures affecting both the upper and lower limbs had significantly more activity limitations and participation restrictions. The same results were also found when comparing elderly residents with contractures isolated to the upper limbs vs. isolated to the lower limbs and those with contractures isolated to the lower limbs vs. affecting both the upper and lower limbs.

Finally, regardless of the contracture category or contracture site, stroke was an important complication. As already noted, the association between spasticity after stroke and joint contracture development is more than a key complication. Previous research perspectives indicate that contractures usually present together with stroke, raising the question of whether they are related [61–63].

Some potential limitations should be considered. First, although the sample size in this study satisfied the requirements for establishing stable person and item estimates and a power analysis, caution is necessary when generalising our results because of the small sample size.

Second, although other diseases associated with joint contractures were addressed (e.g., stroke and diabetes), the study distinguished between joint contractures with and without OA. However, joint contractures are associated with several health conditions, immobility, and aging. Factors that may be potentially associated with activities and participation in the elderly residents (e.g., comorbidity and premorbid lifestyle) warrant scrutiny in future research.

Third, the diagnosis of OA in our study was based on radiographic evidence. Given the advances of technology, quantitative magnetic resonance imaging (qMRI) has been used due to many advantages over radiography and allows the assessment of joint structures, joint space width, and bone shape in 3 dimensions and at high resolution. As a limitation to our research, a diagnosis of OA based on radiography may be less sensitive in detecting early structural changes than MRI measurements.

Finally, chronic diseases were assessed through elderly residents’ self-reports and medical records. This approach may not be as rigorous as standardized diagnostic tests and may have inherent biases that may disadvantage certain groups, such as elderly residents who are unfamiliar with chronic diseases or who are not proficient at relating terms for chronic diseases.

## Conclusions

The activities and participation of elderly residents in LTCFs varied when contracture sites differed. In addition, the explanatory power of contracture sites for activities and participation had a moderate strength of association. Compared with other contracture sites, regardless of OA, contracture sites in both the upper and lower limbs were associated with the greatest activity limitations and participation restrictions. Elderly residents in LTCFs belonging to minority groups, with a history of stroke, and with OA are at a high risk of developing activity limitations and participation restrictions. However, no significant association between joint contractures and QoL among elderly residents was found.

The present study fills the research gap on activity and participation in elderly residents with joint contractures in LTCFs by providing a comprehensive survey of activity limitations and participation in this population. In addition, the results of this study can be used to improve the health care, rehabilitation, prevention, and research in LTCFs. For example, identification of risk factors in the population may facilitate environmental adaptation and formulation of compensatory strategies to encourage activity participation of elderly residents with joint contractures.

This present study also raised a number of issues that are relevant for continued research. For example, what are the differences in activity participation between elderly residents in LTCFs and those in the general community? What are the determinants of change in activity participation in the elderly residents in LTCFs? Are activities performed by elderly residents in LTCFs more likely to be biased toward upper limb involvements to reduce the risk for falling? In clinical practice, activities should be diversified for enriched experiences to conform to the notion of holistic health care in LTCFs.

## Supplementary Information

**Additional file 1.**

## Data Availability

Data from this study are available in the Chinese Clinical Trial Registry; registration number and date: ChiCTR2000039889 (13/11/2020). Hyperlink: http://www.chictr.org.cn/listbycreater.aspx

## Abbreviations

CASI C: Chinese Version of the Cognitive Abilities Screening Instrument
CFI: Comparative fitness index
CI: Confidence interval
COVID-19: Coronavirus disease 2019
EFAs: Exploratory factor analyses
ICF: International Classification of Functioning, Disability, and Health
MDS: Minimum Data Set
LTCF: Long-term care facilities
MMSE: Mini-Mental Status Examination
OA: Osteoarthritis
QoL: Quality of life
ROM: Range of motion
WHODAS 2.0: WHO Disability Assessment Schedule 2.0
WHOQOL-BREF: World Health Organization Quality of Life-BREF
