# Supplementary material for: Associations among quality of life, activities, and participation in elderly residents with joint contractures in long-term care facilities: a cross-sectional study
Source: BMC Geriatr. 2022 Mar 12;22:197. doi: 10.1186/s12877-022-02870-6 (PMC8917858; doi:10.1186/s12877-022-02870-6)
Supplement: Supplementary file 1 — Additional file 1. [file 12877_2022_2870_MOESM1_ESM.docx]

**IMPLICATIONS FOR REHABILITATION**

- Although activity and participation involved many factors, they were found to be the most important domains for the quality of life of older long-term care facility residents.
- Joint contractures are a major cause of activity limitations and participation restrictions in elderly residents in long-term care facilities.
- Minority status, non-mainstream religious beliefs, stroke, and osteoarthritis were the four key risk factors for activity limitations and participation restrictions in elderly residents with joint contractures at long-term care facilities.
- The framework of risk factors for activity limitations and participation restrictions reported in this study may be helpful for medical professionals and in implementing interventions for older patients with contractures.
